# Supplementary material for: Combined Effects of Temperature and Dietary Lipid Level on Body Composition, Growth, and Freshness Profile in European Seabass, Dicentrarchus labrax
Source: Animals (Basel). 2023 Mar 15;13(6):1068. doi: 10.3390/ani13061068 (PMC10044243; doi:10.3390/ani13061068)
Supplement: Supplementary file 1 [file animals-13-01068-s001.zip › animals-2107258-supplementary.pdf]

Supplementary material

Combined effects of temperature and dietary lipid level on body composition, growth and freshness profile in European seabass, *Dicentrarchus labrax*

Patrícia G Cardoso[1*], Odete Gonçalves[1], Thais Cavalheri[1], Vânia E Amorim[1], Weiwei Cao[2], Diogo A. M. Alexandrino[1,3], Zhongjun Jia[2], Maria F Carvalho[1,4], Paulo Vaz-Pires[1,4], Rodrigo Ozório[1]

Table S1 – detailed composition of diet D-4 Alterna 2P.

Content analysis (%), vitamins and additives.

Content Analysis	D- 4 Alterna 2P
Crude Protein	46.0 %
Crude Oils and Fat	16.0 %
Crude Fibre	3.1 %
Crude Ash	6.8 %
Calcium	1.3 %
Phosphorus	1.1 %
Sodium	0.3 %
Vitamins, additives and antioxidants	D- 4 Alterna 2P
Vitamin A	4103.0 Ul/kg
Vitamin D3	769.0 Ul/kg
Iron	41.0 mg/kg
Manganese	15.3 mg/kg
Copper	5.1 mg/kg
Zinc	92.3 mg/kg

Iodine	2.00 mg/kg
Butilhidroxitoluene (BHT)	50.0 mg/kg
Butylated Hydroxyyanisole (BHA)	100.0 mg/kg

Table S2 - detailed composition of diet L-4 Alterna 2P.

Content analysis (%), vitamins and additives.

Content Analysis	L- 4 Alterna 2P
Crude Protein	46.5 %
Crude Oils and Fat	20.0 %
Crude Fibre	3.8 %
Crude Ash	7.2 %
Calcium	1.6 %
Phosphorus	1.2 %
Sodium	0.4 %
**Vitamins, additives and antioxidants**	**L- 4 Alterna 2P**
Vitamin A	4103.0 Ul/kg
Vitamin D3	769.0 Ul/kg
Iron	41.0 mg/kg
Manganese	15.3 mg/kg
Copper	5.1 mg/kg
Zinc	92.3 mg/kg
Iodine	2.00 mg/kg
Butilhidroxitoluene (BHT)	50.0 mg/kg
Butylated Hydroxyyanisole (BHA)	100.0 mg/kg
